# A close-up on the expanding landscape of CD21^–^/low B cells in humans

**DOI:** 10.1093/cei/uxac103

**Published:** 2022-11-16

**Authors:** Inger Gjertsson, Sarah McGrath, Kristoffer Grimstad, Charlotte A Jonsson, Alessandro Camponeschi, Katrin Thorarinsdottir, Inga-Lill Mårtensson

**Affiliations:** Department of Rheumatology and Inflammation Research, Institute of Medicine, Sahlgrenska Academy, University of Gothenburg, Gothenburg 40530, Sweden; Department of Rheumatology and Inflammation Research, Institute of Medicine, Sahlgrenska Academy, University of Gothenburg, Gothenburg 40530, Sweden; Department of Rheumatology and Inflammation Research, Institute of Medicine, Sahlgrenska Academy, University of Gothenburg, Gothenburg 40530, Sweden; School of Bioscience, University of Skövde, Skövde 54128, Sweden; Department of Rheumatology and Inflammation Research, Institute of Medicine, Sahlgrenska Academy, University of Gothenburg, Gothenburg 40530, Sweden; Department of Rheumatology and Inflammation Research, Institute of Medicine, Sahlgrenska Academy, University of Gothenburg, Gothenburg 40530, Sweden; Department of Rheumatology and Inflammation Research, Institute of Medicine, Sahlgrenska Academy, University of Gothenburg, Gothenburg 40530, Sweden; Department of Rheumatology and Inflammation Research, Institute of Medicine, Sahlgrenska Academy, University of Gothenburg, Gothenburg 40530, Sweden

**Keywords:** CD21–/low, memory B cells, exhaustion, age-associated, atypical, autoimmune-associated

## Abstract

Memory B cells (MBCs) are an essential part of our immunological memory. They respond fast upon re-encountering pathogens and can differentiate into plasma cells that secrete protective antibodies. The focus of this review is on MBCs that lack, or express low levels of, CD21, hereafter referred to as CD21–/low. These cells are expanded in peripheral blood with age and during chronic inflammatory conditions such as viral infections, malaria, common variable immunodeficiency, and autoimmune diseases. CD21–/low MBCs have gained significant attention; they produce disease-specific antibodies/autoantibodies and associate with key disease manifestations in some conditions. These cells can be divided into subsets based on classical B-cell and other markers, e.g. CD11c, FcRL4, and Tbet which, over the years, have become hallmarks to identify these cells. This has resulted in different names including age-associated, autoimmune-associated, atypical, tissue-like, tissue-resident, tissue-restricted, exhausted, or simply CD21–/low B cells. It is however unclear whether the expanded ‘CD21–/low’ cells in one condition are equivalent to those in another, whether they express an identical gene signature and whether they have a similar function. Here, we will discuss these issues with the goal to understand whether the CD21–/low B cells are comparable in different conditions.

## Teaser

CD21-/low memory B cells are expanded with age in healthy individuals and in patients with viral infections and autoimmune conditions and are associated with pathogen-specific antibodies as well as key disease manifestations. Depending on condition, CD21-/low memory B cells have been termed age-associated, autoimmune-associated, atypical, tissue-like, tissue-resident, tissue-restricted, exhausted or simply CD21-/low B cells. CD21-/low memory B cells are often CD27-IgD-CD11c+ and in some conditions they express Tbet, in others FcRL4 and in yet others both.

## Graphical Abstract

**Graphical Abstract:**
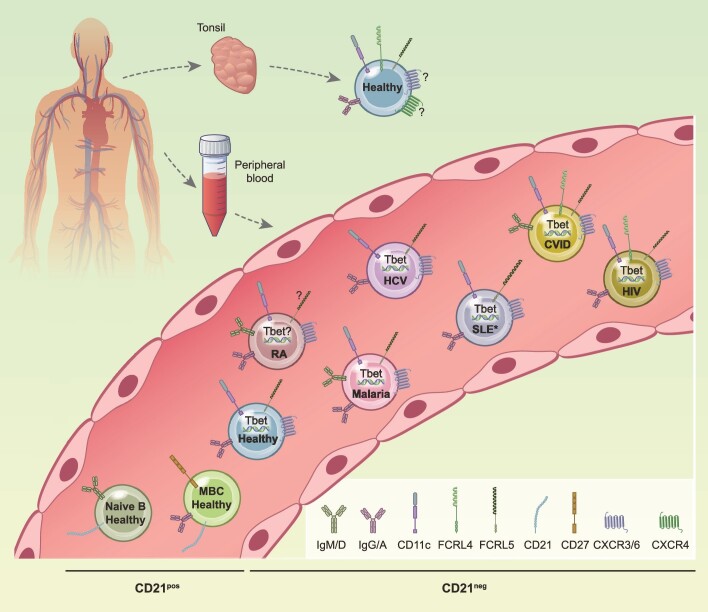

## Introduction

### B-cell populations as defined by classical markers

**Figure 1:**
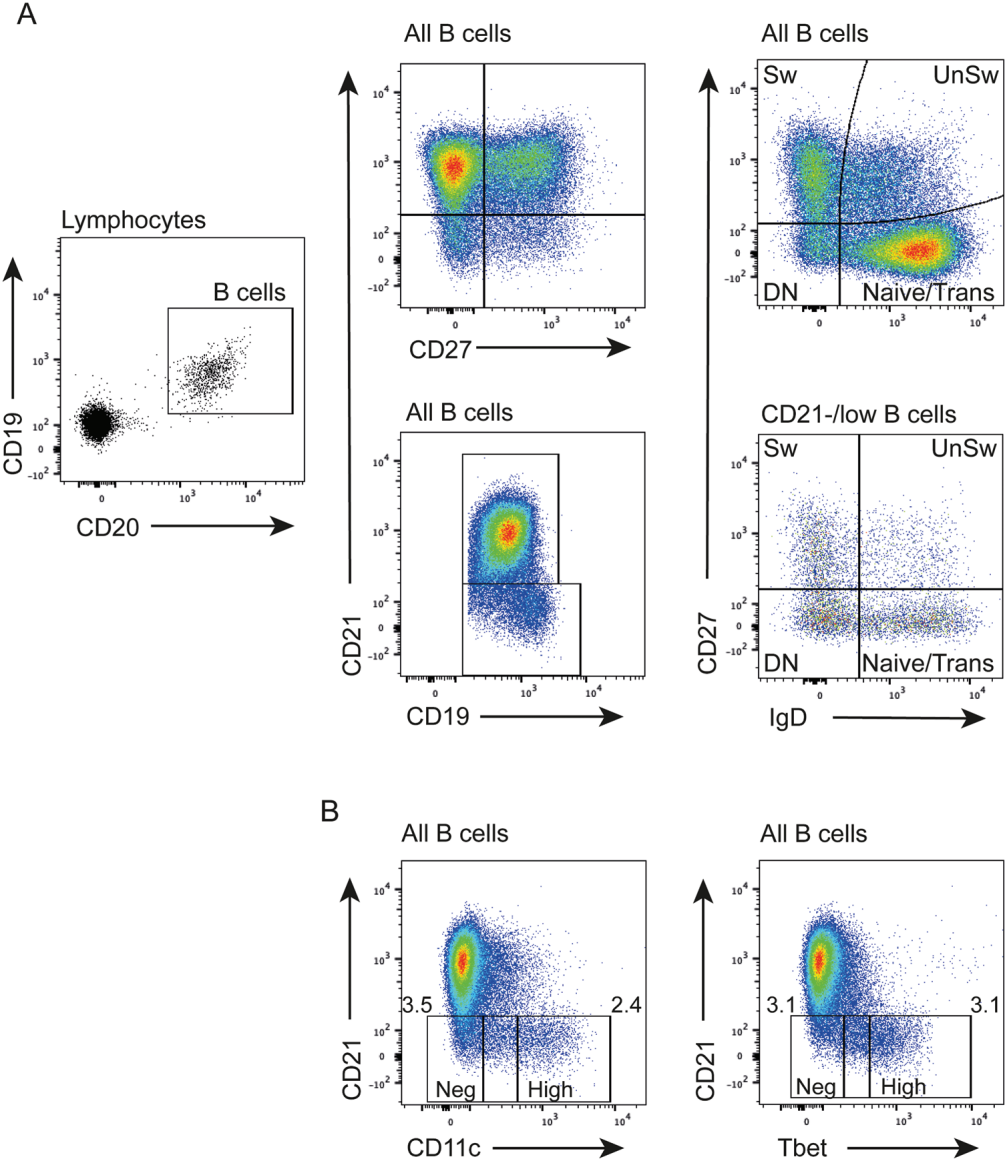
Gating strategies for human B cell subsets. (A) Peripheral blood lymphocytes were gated on CD19+CD20+ and CD19+CD20+CD21–/low B cells, as indicated. Based on CD27 and IgD expression, the cells can be divided into switched memory (Sw), unswitched memory (UnSw), double negative (DN) memory as well as naive and transitional B cells. (B) B cells show heterogeneous expression of CD11c and Tbet, ranging from negative to high levels, showing that high expression levels are largely found on CD21–/low B cells.

B cells can be divided into several populations: transitional B cells, the new emigrants from the bone marrow (BM); naïve B cells that have not yet encountered their cognate antigen; germinal centre (GC) B cells that have encountered antigen and are undergoing proliferative expansion and selection in tertiary structures in secondary lymphoid organs; memory B cells (MBCs) and antibody-producing plasma cells, both elements of immunological memory. Under healthy conditions, circulating peripheral blood (PB) B cells are in a resting state, while the B cells in, e.g. tonsils, representing a lymphoid organ continuously exposed to a plethora of foreign antigens, also contain activated, proliferating B cells. The different B-cell populations can be identified based on their expression of various cell surface markers using flow cytometry. Classical markers that are used to define B cell populations are CD27/IgD [1] and CD24/CD38 [2]. Based on the CD27/IgD markers, MBCs are defined as unswitched (UnSw, CD27+IgD+), switched (Sw, CD27+IgD−), or double-negative (DN, CD27−IgD-) (Fig. 1a). Note that Sw MBCs can be IgM+IgD-. Using the CD24/CD38 markers identifies MBCs as CD38-/low. Over the last decade or so it has become clear that limiting MBCs to only these populations is too simplistic [3, 4]. For instance, although most B cells express CD21 (complement receptor 2 (CR2), a small population of MBCs that express low levels of or altogether lack this marker (herein CD21−/low) have been found. These MBCs are of particular interest because of their increase in frequency with age and during chronic infections and autoimmune inflammatory conditions [5–7], and their association with disease manifestations, as discussed below. The CD21−/low has been found to express exceptionally high levels of the typical B-cell markers CD19 and CD20 [8], and to have a large cell size [9]. Depending on the study, CD21−/low MBCs have been divided into subsets using combinations of the classical B-cell markers and markers such as FcRL4, Tbet, and CD11c. The two latter were initially described to be expressed by CD21−/low age-associated B cells (ABCs) in mice [10, 11]. In this review, we will discuss the phenotype, in vitro responses, gene expression patterns, and association of CD21−/low MBCs with clinical parameters. Our aim is to understand whether the CD21−/low MBCs represent a cell population that shares phenotype and function in health, in autoimmune conditions, and during infections, and whether they are functionally exhausted, as previously proposed.

## CD21−/low B cells in health

### CD21−/low tissue-resident FcRL4+ and tissue-restricted Tbet^hi^ B cells

**Figure 2:**
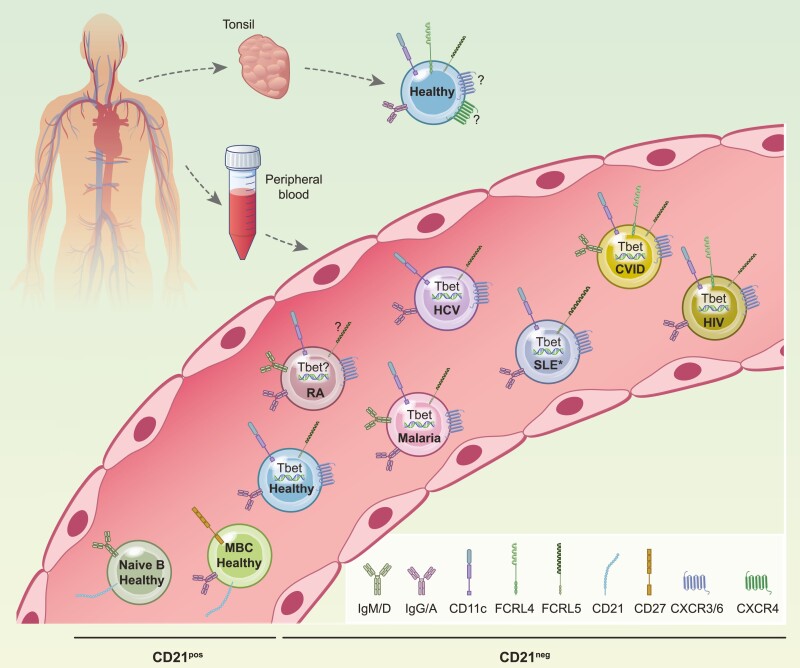
Cartoon depicting phenotypes of CD21–/low memory B cells (MBCs) in tonsils and peripheral blood (PB). In healthy individuals, CD21–/low MBCs in tonsils are FcRL4+, whereas in PB they are Tbet^hi^. The expression of these two markers in PB CD21–/low MBCs in the indicated conditions is shown. Shown is also expression of additional markers according the key. In some conditions, CD21–/low MBCs express different isotypes. Rheumatoid arthritis (RA), hepatitis C virus (HCV), systemic lupus erythematosus (SLE), common variable immunodeficiency (CVID), human immunodeficiency virus (HIV). *In SLE, CD21–/low MBCs have been shown to be FcRL4+ in one study and FcRL4– in another. ? indicates unclear expression.

At the beginning of the 21st century, a new family of immunoglobulin (Ig)-like receptors termed IRTA1, 2, 3, 4, and 5 were discovered [12, 13]. IRTA1 (also named FCRH4; Fc receptor homologue 4 or FcRL4; Fc receptor-like protein) was selectively and consistently expressed by B cells located in the tonsils [9], and in Peyer’s patches [14]. The tonsillar FcRH4+ (hereafter FcRL4+) did not express CD21, and despite lacking CD27, the conventional MBC marker, they had already class-switched to IgG or IgA [9] (Table 1, Fig. 2). Typical of MBCs, the FcRL4+ cells expressed *Ig V* genes that showed signs of somatic hypermutation, in fact on par with the FcRL4− MBCs. In contrast to the FcRL4− cells, those that were FcRL4+ were large in size, consistent with a proliferating population, with an extensive cytoplasm but no rough endoplasmic reticulum, as would be typical of plasmablasts/cells. The FcRL4+ cells were thus considered MBCs and were subsequently found to also express CD11c (αX) integrin and high levels of CD20 [15]. They represented around 10% of tonsillar B cells but were not detected (<0.5%) among PB, BM, or splenic B cells. This led to the conclusion that the FcRL4+ B cells represent a distinct tissue-based population of MBCs. To summarize the phenotype of these tissue-resident CD21−/low MBCs, they are CD27−IgD−CD38−CD11c+FcRL4+. Despite the lack of FcRL4+ B cells in PB from healthy individuals [9], there is a CD21−/low MBC population in PB that constitutes approximately 5% of the B-cell pool, comprising both CD27+ and CD27− as well as switched and unswitched cells [51]. In fact, a recent study showed that in healthy individuals, CD21−/low MBCs are found not only in PB and the tonsils but also in lymph nodes (LNs), spleen, BM, and the thoracic duct [19].

**Table 1:** CD21–/low MBCs; phenotypes, FcRL4 and/or Tbet expression, associations to disease

Condition	Termed	Location	Phenotype in PB	FcRL4	Tbet	Proportion in PB (range)	Disease association	Ref
**Health**								
Healthy individuals	Tissue-resident	Tonsil	CD27−IgD−CD38− CD11c+	**+**	**–**	ND	NA	[9, 15–18]
Healthy individuals	Tissue restricted	PB BM Spleen	CD27+/−IgD− CD38lowCD11c+	**–**	Tbet^hi^	~3% (1–30)^*^	NA	[17, 19, 20]
CVID	CD21**–**/low	PB SLO BAL	CD27−IgD+ IgM+CD38lowCD11c+	**+**	Tbet^hi^	CVID: ~14%	Part of classification criteria	[8, 18, 21–23]
SLE	CD11^hi^ Tbet^hi^	PB Kidneys	CD27− CD38lowCD11c^hi^	**+**	Tbet^hi^	~10% (1–50)	Disease activity, auto ab, PCs	[16]
SLE	DN2	PB	CD27− IgD- CD11c+ CXCR5-	**–**	Tbet^hi^	~20% (2–80)	Disease activity, auto ab, PCs	[24]
Established RA	CD21**–**/low	PB SF	CD27− IgD-	**–**	ND	~10% (2–30)	Joint destruction	[25]
Malaria (*P. falciparum* and *P. vivax)*	Atypical	PB	CD27−CD11c+ CXCR5−	**+/ -**	Tbet^hi^	parasitemia: ~15% (3–21) w/o parasitemia: ~10% (8-12)	Antigen-specific ab, auto ab to red blood cells, and anaemia	[26–36]
HIV	Exhausted, tissue-like	PB	CD27− CD11c+	**+**	Tbet^hi^	~7% (2–22)	HIV-specific IgG	[20, 37–39]
Primary Sjögren syndrome		PB	CD27− CD38lowCD11c+	ND	Tbet+	~10% (1–-25)	ND	[40]
Systemic sclerosis		PB	CD38lowCD11c+	ND	ND	Before treatment: ~5% (1–25) After treatment: ~8% (5–25)	Disease activity	[41, 42]
Crohn’s disease		PB Gut	Tbet+	ND	Tbet+	Active disease: ~3% (2–4) Quiescent disease: ~2% (1.5–3)	Disease activity	[43]
Multiple sclerosis	CD21low	PB Cerebrospinal fluid	CD11c+	ND	ND	<60 years ~1.5% (0–15) >60 years ~1.5% (0–3)	ND	[44]
HBV	Atypical	PB Liver	CD27− CD11c+	ND	Tbet^hi^	~6% (0–16)	HBV specific IgG	[45]
HCV	Tissue-like	PB	CD27−CD11c+	**+**	Tbet+	Non-cirrhosis: ~18% (5–30) Cirrhosis: ~17% (4–70)	Frequency decreases after treatment	[46, 47]
COVID-19	Atypical/DN2	PB	CD27−CD11c+	ND	Tbet+	Mild infection ~15% (1–40) ICU: ~30% (5–75)	Disease activity	[48–50]

Antibody (ab), bone marrow (BM), bronchioalveolar lavage (BAL), cerebrospinal fluid (CSF), common variable immunodeficiency (CVID), double negative 2 (DN2), healthy individuals (HI), hepatitis B virus (HBV), hepatitis C virus (HCV), intensive care unit (ICU), multiple sclerosis (MS), not determined (ND). Not applicable (NA), peripheral blood (PB), rheumatoid arthritis (RA), secondary lymphoid organs (SLO), systemic lupus erythematosus, SLE), Systemic lupus erythematosus disease activity index (SLEDAI), Synovial fluid (SF), Switched.

^*^This is the general level combining all studies in the table.

In mice, splenic CD21–/low ABCs expand with age [11, 52]. These cells express CD11c as well as Tbet [10], a transcription factor first described in T cells as a master regulator of T helper 1 (T_H_1) cell development [53]. It was recently reported that CD11c and Tbet are also expressed in human PB CD21–/low B cells [16, 19, 24]. Consistent with these reports, the flow cytometry plots in Fig. 1b show that PB CD21–/low MBCs express Tbet and CD11c, and that the cells can be divided into different subsets based on expression levels. Moreover, CD21–/lowTbet^hi^ cells are also present in spleen and BM, constituting on average 3–4% of all B cells in the respective tissue, though proportions varied between donors, from 1% to 10% [19]. Due to the lack of CD21–/lowTbet^hi^ MBCs in LNs and thoracic duct, this suggests that these cells do not circulate via the lymphatic system. The CD21–/lowTbet^hi^ cells were, therefore, referred to as tissue-restricted MBCs. Among the splenic CD21–/lowTbet^hi^ MBCs were antigen-experienced cells that expressed influenza-specific IgG1 on their cell surface [19], suggesting that these cells derive from previous influenza infections. The presence of CD21–/lowTbet^hi^ in PB and spleen and their absence in LNs has been confirmed in other studies where it was also found that CD21–/lowTbet^hi^ cells are not present in tonsils [17]. To summarize the phenotype of the tissue-restricted CD21–/low MBCs, they are CD27+/–CD38lowCD11c+Tbet^hi^.

The discovery of a novel FcRL4+ MBC population in tonsils led to the analyses of this marker on CD21–/low B-cells expanded in chronic inflammatory conditions. While FcRL4+ MBCs were found in tonsils but not PB in healthy individuals, the situation might be different in disease, which turned out to be the case in common variable immunodeficiency (CVID) and human immunodeficiency virus (HIV). The discovery of CD21–/lowTbet^hi^ MBCs in healthy PB revealed that the expression of FcRL4 and Tbet^hi^ appears to be mutually exclusive. However, in CVID and HIV CD21–/low MBCs are FcRL4+Tbet^hi^ while in other autoimmune conditions and most chronic infections the CD21–/low MBCs are FcRL4-Tbet^hi^, as discussed below.

## CD21–/low B cells and associations under chronic inflammatory conditions

Compared with healthy individuals, an expansion of PB CD21–/low B cells has been described in chronic inflammatory conditions. In these disease contexts, CD21–/low B cells have been termed ABCs (by analogy to these cells in mice), atypical (malaria), tissue-like and/or exhausted (HIV), autoimmune-associated or, simply, CD21–/low B cells. In some conditions, not only PB but also additional tissues have been analysed, e.g. spleen, LN, BM, kidneys, and synovial fluid/tissue. The initial studies of CD21–/low MBCs did not assess the expression of Tbet and/or FcRL4. However, based on classical B-cell markers and Ig isotypes, we link the early and more recent studies to aid our understanding of CD21–/low MBCs in chronic inflammatory conditions.

### Common variable immunodeficiency

CVID is one of the more common primary immunodeficiencies. The aetiology is unknown, but an increasing number of different genetic mutations have been discovered. At large, CVID is a B-cell disease and is characterized by low levels of Igs. However, disease manifestations, severity, and expressed Ig isotypes vary between individuals, hence the description ‘variable’. Already in 2002, a group of CVID patients was found to present with an expanded PB CD21–/low B-cell population, in particular patients more likely to have splenomegaly, interstitial lung disease, and other autoimmune complications [8]. The CD21–/low cells were CD27–CD38lowCD11c+ and expressed unmutated IgM and IgD, although this may reflect an inability to class-switch or form functional GCs, depending on the CVID genotype [21]. In addition to FcRL4, the CD21–/low cells expressed FcRL5, another Fc receptor-like protein. The view that there is an expansion of CD21–/lowCD27– B cells in CVID is supported by others [54]. More recently, PB CD21–/low B cells in CVID patients were found to be Tbet^hi^. These CD21-/lowTbet^hi^ cells are also present in secondary lymphoid organs (SLOs) and spleen, and in bronchoalveolar lavage (BAL) in CVID patients with interstitial lung disease [17, 18]. Taken together, CD21-/low B cells in CVID are CD27–IgM+IgD+CD38lowCD11c+FcRL5+FcRL4+Tbet^hi^, and depending on the patient-specific genetic defect, CD21–/low B cells are present in spleen, BAL, and SLOs. Therefore, the PB CD21–/low B cells in CVID differ phenotypically from those in healthy individuals in that they are not only Tbet^hi^ but also express FcRL4. That the latter is a marker for tissue-residency seemingly does not apply in CVID.

## Autoimmune diseases

Autoimmune diseases affect approximately 5–7% of the human population. In most cases the aetiology is unknown, although sex, genetics, and environmental factors are known to play important roles. These diseases are heterogeneous and include a wide range of disorders such as systemic lupus erythematosus (SLE), rheumatoid arthritis (RA), systemic sclerosis, and Crohn’s disease. Each diagnosis is based on multiple criteria, which means that there can be considerable variation between individuals.

### Systemic lupus erythematosus

SLE is a rheumatic disease that primarily affects younger women and can involve almost every organ in the body including blood, skin, and kidneys. The inflammation is typically mediated by immune complexes that are deposited in e.g. the kidneys, which contributes to the development of nephritis. The proportions of various B-cell populations in SLE patients differ depending on disease manifestation and activity, measured using the Systemic Lupus Erythematosus Disease Activity Index (SLEDAI) [55–57]. An expansion of CD21–/low B cells has been described in SLE patients, where this cell population was identified as CD27+/–CD38low expressing IgM or IgG, and the frequency of these cells correlates with disease duration and SLEDAI score [8, 58, 59]. Several groups have further defined the CD21–/low MBCs in PB that are expanded as CD27–CD38lowCD11c^hi^FcRL5+FcRL4+Tbet^hi^, switched or unswitched, or CD27–IgD–CD38lowCD11c+CXCR5–FcRL5+FcRL4–Tbet^hi^ (also termed double negative (DN2) cells). In both studies, the frequency of these cells is associated with SLEDAI score and autoantibody levels [16, 24], results supported by others [60, 61]. CD21–/lowCD27–CD38^low^CD11c^hi^FcRL5+FcRL4+Tbet^hi^ MBCs are also associated with clinical manifestations such as malar rash and nephritis, and CD11c+ B cells have been found in nephritic kidneys [16], although the status of CD21 and CD27 is unclear. CD21–/lowTbet^hi^ cells have also been found to associate with increased serum levels of interferon-gamma (IFNγ) as well as interferon-lambda (IFNλ) [60, 61].

There is therefore ample evidence that CD21–/lowTbet^hi^ MBCs are expanded in SLE patients, and at frequencies that correlate with both disease activity and serum levels of typical lupus autoantibodies, e.g. anti-dsDNA, anti-Smith antigen and anti-RNP. Furthermore, SLE studies from different laboratories have identified a relatively consistent phenotype, although FcRL4 expression is less clear, the CD21–/low MBCs that associate with disease manifestations are CD27–CD38^low^CD11c+CXCR5–FcRL5+Tbet^hi^.

### Rheumatoid arthritis

Rheumatoid arthritis (RA) is the most common inflammatory rheumatic disease that leads to synovitis and subsequent joint destruction, most commonly in the hands and feet. During the last decade, it has become clear that B cells are one of the major players in this disease as B-cell depletion with rituximab (anti-CD20 antibodies) can induce remission, particularly in patients positive for antibodies reactive with citrullinated proteins (anti-citrullinated protein antibodies (ACPAs)) and/or Igs, i.e. rheumatoid factor (RF). There are several studies that have described an expansion of CD21–/low B cells in PB from patients with established RA. However, the markers vary, for instance, the expanded CD21–/low B cells have been described as CD27–CD38– [54], CD38– [62], CD27–IgD–IgM– [63]. Although CD21–/low B cells have been shown to express Tbet at the mRNA level [54], it is unclear whether it is all CD21–/low cells or a subset thereof. In ACPA^+^/RF^+^ patients with established RA, CD21–/lowCD27–IgD–FcRL4– MBCs are expanded, and the frequencies of these cells correlate with joint destruction [25]. In another study, CD11c+Tbet+ B cells (of unknown CD21 and CD27 status) were found to associate with inflammation and disease activity (disease activity score 28 joints (DAS28)) [64]. Moreover, the frequency of CD21–/low that are CD27+ was increased in female RA patients and was found to correlate with age [11].

In RA, the synovium is the main site of inflammation. The type of local inflammation can vary between patients, with patients stratified into three categories based on immune cell infiltration: pauci-immune, diffuse-myeloid, and lympho-myeloid [65]. Among these, lympho-myeloid synovium harbours a substantial number of infiltrating B and T cells and can be further divided into B cell-rich and B cell-poor based on the number of B cells and B cell-aggregates detected by immunohistochemistry [66]. It has been suggested that B cell-rich synovitis is associated with high disease activity [65]. CD11c+Tbet+ B cells have been found in synovial tissue sections [67], although the CD21 and CD27 status is unclear. In synovial fluid, although B-cell frequencies are low, the vast majority of these are CD21–/low [68], and most of these are CD11c+IgD–, some of which are CD27–FcRL4+ [25]. The presence of FcRL4+ B cells in synovial tissue is supported by other studies [69]. Others have shown a high abundance of CD21–/lowCD11c+Tbet+ MBCs, although the proportions varied (1–90%) between donors [70]. Thus, the expanded CD21–/low MBCs in PB in RA are associated with disease manifestations are CD27–IgD–FcRL4–.

## Other autoimmune diseases

CD21–/low B cells are also expanded in patients with other autoimmune diseases. Below, we briefly summarize their phenotypes and associations with clinical manifestations. In patients with *systemic sclerosis*, a rare but severe rheumatic disease characterized by Raynaud’s phenomenon, inflammation and fibrosis of skin, blood vessels and internal organs, the expanded CD21–/low cells are CD38low, and half of them are CD11c+ and switched [41, 42, 71]. In patients with the highest frequencies of CD21–/lowCD38low, these are associated with disease manifestations both at diagnosis and over time. In patients with *primary Sjögren’s syndrome*, an inflammatory rheumatic disease characterized by sicca symptoms and an increased risk of developing lymphoproliferative disease, the expanded CD21–/low MBCs have been described as CD27–CD38low, most were IgM+IgD+ and mutated, whereas only a small (approx. 20%) proportion expressed Tbet or CD11c [40, 72]. Further, CD21–/lowCD11c+IgM+ and IgG+ MBCs are expanded in *multiple sclerosis* (*MS*), an autoimmune inflammatory disease that engages the brain and spinal cord [44]. The frequency of these cells has been found to be higher in cerebrospinal fluid compared with those in PB. *Crohn’s disease* is a chronic inflammatory bowel disease that can affect the entire gut. In patients with Crohn’s disease, Tbet+IgG+ MBCs (CD21 status unknown) are expanded in both PB and gut (ileum) and correlate with disease activity [43]. Taken together, in all these diseases a CD21–/low population is expanded, except for Crohn’s where the expanded cells were defined as CD19+Tbet+. The expression (or not) of FcRL4 is unclear.

## Acute and chronic infections

### Malaria

Malaria is caused by protozoans belonging to the *Plasmodium* family including *P. falciparum* and *P. vivax*. The infection is characterized by high fever and shaking chills; it has variable severity from mild to severe organ manifestations such as cerebral malaria and anaemia. An expansion of PB CD21–/lowCD27–CD11c+, mainly IgG+, MBCs have been described in children persistently exposed to malaria [73] and individuals in malaria-endemic areas [26, 27]. Post-treatment, as patients become free from parasites, they no longer present with high CD21–/low B-cell frequencies [74]. The immune response to *P. falciparum* infections is dynamic, as observed in acutely infected and previously exposed individuals, where mainly CD21–/low CD11c+ B cells are first expanded and then decrease as the infection resolves [28]. Among CD21–/lowCD11c+CD27–, those that are IgD+ are expanded in the primary response and both IgD+ and IgD– are increased in the recall response. These dynamics of the B-cell response during acute malaria were confirmed in another longitudinal study [29]. Moreover, antigen-specific B cells are largely CD21–/lowCD11c+, and neutralizing IgG antibodies to *P. falciparum* are produced by CD21–/lowCD27– MBCs [29, 30]. The mutation status of these antibodies is equivalent to antibodies derived from classical MBCs [30, 31]. Most of the PB CD21–/lowCD27– cells are CD11c+CXCR5–FcRL5+FcRL4–Tbet^hi^ and switched (IgG1, IgG3) [32, 75]. In some patients with acute malaria caused by *P. falciparum* or *P. vivax*, serum levels of autoantibodies against phosphatidylserine (which is exposed on the surface of dying erythrocytes) and red blood cells correlate with PB Tbet^hi^ B cells and the development of anaemia [33, 34]. Thus, in malaria, the expansion of CD21–/low MBCs is associated with infection phase and represents cells that give rise to antigen-specific and autoantibodies where the latter can result in anaemia.

### COVID-19

The last few years have been dominated by the COVID-19 pandemic, and much effort has been put into understanding the immunological mechanisms and finding useful strategies to combat the virus. In critically ill COVID-19 patients CD21–/lowCD27– B cells have been observed to be expanded compared with healthy individuals [48]. This observation was confirmed by another study reporting an expansion of CD21–/lowCD27–IgD–CD38–CD11c+Tbet^+/hi^ in the critically ill [49]. The number and proportion of CD21–/lowCD27– cells decreased in patients who recovered but remained expanded in patients who succumbed to the disease [50]. Compared with vaccination-induced MBCs, those induced by the infection showed better antigen-binding capacity and generated more CD21–CD27–CD11c+ MBCs [76].

### Human immunodeficiency virus

HIV infects immune cells, preferentially CD4^+^ T cells, and with antiretroviral therapy, patients live with chronic infection. Although the T cells are the main viral target there are also changes in the peripheral B-cell compartment in these patients [77]. PB CD21–/lowCD27–CD11c+FcRL4+ B cells appear in association with viraemia, are more frequent in viraemic compared with aviraemic patients, produce HIV-specific antibodies, and decrease with treatment [37, 38]. A few years ago, a study found that during HIV infections the specific HIV gp140 response is dominated by expanded PB CD21–/lowCD27–CD11c+Tbet^hi^, switched, B cells [20]. These cells were found to express both FcRL4 and FcRL5 at the mRNA level. More recent work, analysing LNs, showed that HIV-specific B cells of infected individuals were enriched among CD19^hi^Tbet^hi^ MBCs and that they expressed mutated Ig genes [39]. The CD21–/low B cells in LNs of HIV patients were Tbet^hi^ whereas no such cells were detectable in LNs from healthy individuals. In conclusion, the expanded CD21–/low B cells in PB from HIV patients are Tbet^hi^FcRL4+, and hence similar to those in CVID.

### Hepatitis B and C

Hepatitis B virus (HBV) can cause chronic infection and cirrhosis, putting the infected individual at high risk of liver cancer and death. HBV infection is also linked to the development of a vascular autoimmune manifestation, polyarteritis nodosa. During chronic HBV infection, PB CD21–/lowCD27–CD11c+FcRL5+Tbet^hi^, largely switched MBCs are expanded and constitute approximately 30% of HB-specific cells, in contrast to HBV-vaccinated patients where the proportion is 10% [45]. Moreover, decreased viral load is associated with decreased proportions of CD21–/low MBCs.

Until recently, hepatitis C virus (HCV) infection was also considered a chronic disease, strongly associated with cirrhosis and hepatocellular cancer. Now, direct-acting antivirals clear the infection within 8–12 weeks in approximately 90% of patients. In HCV-infected individuals, both with and without cirrhosis, there is an expansion of PB CD21–/lowCD27– B cells, of which most lack FcRL4 [46]. Tbet+ B cells are also expanded in HCV-infected individuals; most of these cells were CD21–/lowCD27–CD11c+FcRL5+ and had switched to IgG [47]. Treatment and resolution of infection reduce the levels of the Tbet+ B cells.

## Conclusion on phenotype of the expanded CD21–/low MBCs

Taken together, the phenotype that emerges for the expanded PB CD21–/low MBCs can, in most conditions, be defined as CD27–IgD–CD11c+ and largely switched. In malaria and HCV the cells are FcRL4–Tbet^hi^, in CVID and HIV they are FcRL4+Tbet^hi^, and in SLE they are Tbet^hi^ while the expression of FcRL4 is unclear. In RA the cells are FcRL4– whereas their Tbet expression is unclear. Therefore, in SLE, malaria and HCV, and likely in RA, but not in CVID and HIV, the expression of FcRL4 and Tbet^hi^ is similar to that in healthy individuals. In infectious conditions, the expansion of CD21–/low MBCs is associated with the viral or parasitic load and there are indications that these cells contribute to the production of both disease-specific antibodies and pathogenic autoantibodies. In autoimmune diseases, these cells are associated with key disease manifestations and autoantibodies.

## CD21–/low MBCs and exhaustion

A decade ago, the CD21–/low B cells in HIV were proposed to represent a population of exhausted B cells [38]. Exhaustion, or cell exhaustion, is a differentiation state in which cells become unresponsive to stimulation. This phenomenon was first described for T cells in lymphocytic choriomeningitis virus (LCMV) infections and subsequently also in HIV [78]. The conditions that drive exhaustion—chronic antigen exposure and T-cell receptor stimulation—occur in cancer, chronic infection, and autoimmunity. Exhaustion encompasses reduced proliferative potential, increased expression of inhibitory receptor, and enhanced epigenetic enforcement [27, 75, 79, 80]. Upon analysis of the CD21–/lowCD27– B cells in HIV, it was found that these cells responded poorly to BCR activation in vitro and displayed increased expression of inhibitory receptors, e.g. FcRL4 [38]. Similar findings were evident in malaria-infected individuals where the response of CD21–/lowCD27– B cells was poor with respect to proliferation, cytokine production, and production of specific antibodies [27, 75]. However, in malaria the CD21–/low cells are not considered exhausted, as the response of these cells seemingly depends on the type of stimuli, in fact, these cells might even have a beneficial role [81].

### In vitro proliferation and differentiation capacity

One of the criteria for considering the CD21–/lowCD27– MBCs in HIV as exhausted was their reduced in vitro proliferative response to signaling through the BCR (anti-Ig), although they did respond to anti-Ig when combined with TLR9 agonist (CpG) and CD40L, on par with classical MBCs [38] (Table 2). Moreover, after polyclonal stimulation (*Staphylococcus aureus* and CpG) the cells differentiated into plasma cells producing HIV-specific antibodies. In CVID, compared to naïve B cells, the CD21–/low cells show reduced proliferation in response to anti-Ig combined with CD40L and cytokine, whereas they secrete more antibodies after stimulation with CD40L and cytokines [21]. Somewhat similar, in SLE, CD21–/low MBCs isolated as DN2 cells or CD11c^hi^ showed a poor proliferative response after activation with TLR7 combined with cytokines or CD3-activated T cells, whereas generation of Ig secreting cells was comparable with classical MBCs [16, 24]. Moreover, the CD21–/low MBCs produced typical SLE autoantibodies. Also in malaria, CD21–/lowCD27– MBCs show a poor proliferative response to anti-Ig combined with anti-CD40, CpG, and cytokines [75]. These stimuli did not give rise to antibody production. However, when stimulated with SEB and autologous Tfh cells, the cells differentiated into plasmablasts and secrete antibodies, although not to the same extent as classical MBCs [29]. In RA, CD21–/low MBCs proliferate and differentiate in response to anti-Ig combined with TLR7 agonist and cytokine, on par with classical MBCs [25]. When it comes to PB CD21–/low MBCs from healthy individuals, although their proliferative response to TLR7 agonist and cytokines was reduced compared to classical MBCs, combining with anti-Ig resulted in a response as efficient as classical MBCs in terms of both proliferation and plasmablast differentiation [51]. The study on tonsillar FcRL4+ (CD21–/low) MBCs found that their proliferative response to anti-Ig was poor whereas CD40L combined with cytokines induced proliferation and generated antibody secreting cells, similar to classical MBCs (FcRL4–) [9]. Together this shows that the CD21–/low MBCs, whether the cells are obtained from healthy individuals or from those with chronic inflammatory conditions, can be induced to differentiate into plasma cells secreting (auto) antibodies, similar to classical MBCs. The proliferative response of the CD21–/low MBCs, however, varies and might depend on their transcriptome, as discussed below, but also their in vivo experience, as the cells are large in size and at least some of them have undergone expansion (Ki-67+) [15, 20, 28] [30, 82].

**Table 2:** In vitro activation of CD21–/low MBCs

Condition	CD21–/low phenotype	Control cells	Proliferation (stimuli)	ASC/Ig secretion (stimuli)	Ref
**DISEASE (peripheral blood)**					
HIV	CD21– CD27–	CD27+ MBCs	Reduced (anti-Ig+CD40L), similar (anti-Ig + CD40L+ CpG)	Reduced frequency of ASC and increased frequency of HIV-specific ASC (*S. aureus* + CpG)	[38]
CVID	CD21-/low	Naïve B cells	Reduced (anti-Ig+cytokine+CD40L or CpG stimulation)	ND/ increased (cytokine+CD40L)	[21]
SLE	CD11c^hi^	Classical MBCs	ND	Similar/ similar (Co-culture with anti-CD3 activated T cells)	[16]
SLE	CD21-/low CD11c+ Tbet^hi^ (DN2)	Classical MBCs	Reduced (R848 + cytokines)	Similar (R848 + cytokines)	[24]
Malaria	CD21–CD27–	Classical MBCs	Reduced (anti-Ig+ anti-CD40+cytokines+CPG)	None/ND (CpG, IL-10, SAC and PWM)	[75]
Malaria	CD21– CD27– MBCs	Classical MBCs	ND	Reduced/ ND (SEB + cTfh cells)	[29]
Established RA	CD21–/low	Classical MBCs	Similar (anti-Ig+ R848+ cytokine)	Similar/ ND (anti-Ig+ R848+ cytokine)	[25]
**HEALTH**					
PB	CD21–/low MBCs	Classical MBCs	Reduced (R848+IL2) similar (anti-Ig+ cytokine+ R848)	Similar/ ND (anti-Ig+ cytokine+ R848)	[51]
Tonsils	(CD21–/low) FcRL4+ MBCs	Classical FcRL4– MBCs	Reduced (anti-Ig), similar (cytokines+CD40L)	Increased/ND (cytokines + CD40L)	[9]

Antibody secreting cells (ASC), anti-Ig (anti-Immunoglobulin), common variable immunodeficiency (CVID), double negative 2 (DN2), human immunodeficiency virus (HIV), not determined (n/d), peripheral blood (PB), pokeweed mitogen (PWM), rheumatoid arthritis (RA), Staphylococcus aureus Cowan (SAC), Staphylococcus enterotoxin B (SEB), Systemic lupus erythematosus (SLE), CpG Toll like receptor 9 agonist, R848, Toll like receptor 7/8 agonist.

### Expression of inhibitory receptors

As discussed above, the CD21–/low MBCs in HIV express FcRL4 [38], which has been considered to be an inhibitory receptor due to its immunoreceptor tyrosine-based inhibitory motifs (ITIMs). Indeed, signalling via FcRL4 in the context of BCR activation is inhibitory; however, in the context of Toll-like receptor (TLR) agonists it is activating [83]. Like FcRL4, FcRL5 is a member of the Fc receptor-like proteins and has immunomodulatory potential [84], and is also expressed on CD21–/low MBCs in HIV [20]. Unlike FcRL4, which is a receptor for IgA, FcRL5 binds IgG [85]. FcRL5 has both inhibitory (ITIMs) and activating motifs (ITAMs), implying inhibitory and stimulatory potential. Recent work suggests that FcRL5 is part of the BCR co-receptor complex, together with CD21, and amplifies the BCR response [84]. Hence, under these conditions FcRL5 has activating properties. However, in the absence of CD21, FcRL5 acts as an inhibitory receptor and reduces the response to BCR stimulation. This does not necessarily mean that the expression of FcRL5 is synonymous with unresponsiveness, as this may depend on stimuli, although this is less clear. In the context of CD21–/low MBCs, in SLE, malaria, HCV, and RA they lack FcRL4, whereas in CVID and HIV they express this receptor. FcRL5 is expressed on CD21–/low MBCs in most conditions. As the function of FcRL4 and FcRL5 is seemingly dependent on the stimuli, expression of these receptors is not necessarily a sign of exhaustion.

## Chemokine receptor-expression

Cells in PB that express CXCR3 and/or CXCR6 migrate towards sites of inflammation and/or extralymphatic sites. By contrast, expression of CXCR4 indicates migration to BM, spleen and LN, and expression of CXCR5 to spleen and LNs. These chemokine receptors can also be an indication of where cells reside. A pattern of CXCR3/CXCR6 expression and a lack of CXCR4 and CXCR5 has emerged for CD21–/low MBCs, which appears to be independent of condition and of whether the cells express FcRL4, FcRL5, and/or Tbet (Table 1). This said, this does not mean that all CD21–/low MBCs that are for instance Tbet^hi^ express CXCR3. Rather, expression of these receptors is seemingly dependent on disease dynamics, as exemplified in malaria [28]. Here, the proportion of CXCR3 positive CD21–/lowCD27–CD11c+ MBCs increased from around 20% to 70% during the first month after primary infection. Nevertheless, this suggests that the CD21–/low MBCs in PB that express CXCR3/CXCR6 are destined for inflammatory sites.

## Gene expression in CD21–/low MBCs

Several laboratories have analysed genes expressed by the CD21–/low B cells to understand their fate, for instance, whether they are on a path to plasma cells, as they in all conditions can differentiate into antibody secreting cells in vitro. In general, these studies corroborated the expression (or lack) of identifying phenotypic markers at the mRNA level, e.g. expression of *ITGAX* (CD11c), *TBX21* (Tbet), and/or *FCRL4, FCRL5,* and *CXCR3/6*, but lack of *CR2*, *CD27,* and *CXCR4/5*. Below we discuss genes that are shared, and others that characterize the CD21–/low MBCs in different conditions.

Analysing sorted CD27–IgD– PB B cells from healthy individuals by single-cell RNA sequencing (sc-RNAseq) identified four clusters (DN1-4), of which DN2 likely represented the CD21–/lowCD27–IgD– MBCs, expressing *ITGAX*, whereas DN1 and 4 showed an expression pattern similar to classical MBCs [3]. Other genes expressed in DN2 included *TBX21*, although not all cells were positive, *FCRL5* and *TNFRSF1B* but not *CXCR5, IL4R*, *IGHM* or *IGHD*. The expression or lack of these genes is confirmed in another sc-RNAseq study [86]. Genes indicative of plasma cell precursors were not discussed, presumably not expressed. Analysing tonsillar CD21–/lowFcRL4+ MBCs detected *AICDA* (AID) but not genes typical of plasma cells, e.g. *PRDM1* (BLIMP1), *IRF4*, and *XBP1* [9, 15], which indicated that neither of these cells are plasma cell precursors.

In HIV, antigen-specific PB CD21–/lowCD27– IgG+ MBCs were found to express *AICDA* and *TNFRSF1B* [87], which has been confirmed by others in sc-RNAseq data [35]. The latter study also detected high levels of *SYK* and a lack of *IL4R*. Isolating CD19^hi^ HIV-specific MBCs and, by inference, CD21–/lowTbet+FCRL4+, from LNs enabled the identification of *AICDA* [39]. High levels of *SYK* mRNA have also been detected in PB CD21–/low MBCs in CVID [88]. Analysis of PB CD21–/lowCD27– MBCs as bulk or the corresponding population in sc-RNAseq data from patients in a malaria-endemic area found high levels of *AICDA*, *SYK*, *TNFRSF1B* as well as *IL21R*, and IFNγ signature [35, 89]. In the sc-RNAseq study the expression of plasma cell genes, e.g. *PRDM1* was prominent in the ‘activated MBC’ but not in the ‘ABC’ (CD21–/low) cluster. Another study followed the dynamics of malaria-specific Tbet-expressing CD21–/lowCD27– B-cell responses to acute malaria infection [29]. It demonstrated that *IL21R* was expressed in healthy individuals in malaria-endemic areas before the start of the malaria season and down-regulated a week after treatment of the first acute malaria episode (in convalescence), while *TLR7*, *TLR9*, *PRDM1,* and *CD38* showed the opposite pattern. These findings indicate that at convalescence the CD21–/lowCD27– are plasma cell precursors.

In SLE, isolated PB CD11c^hi^ and, by inference, CD21–/lowTbet^hi^ MBCs were found to express high levels of *AICDA, TNFRSF1B*, *SYK*, *IL21R* as well as *TLR9* and *TLR7*, and low *IL4R* [16]. Pathway analyses found an enrichment of IL-21-inducible genes. Moreover, the cells expressed genes indicative of plasma cell precursors, e.g. *PRDM1* and *XBP1* [16]. Similar gene expression patterns were found in another SLE study of PB CD21–/low DN2 (CD27–IgD–CD11c+CXCR5–Tbet^hi^) MBCs, for instance, *PRDM1* and *XBP1* but also *IRF4* [24]. Moreover, these cells expressed elevated levels of *IFNLR1* (IFNλ receptor 1), supported by others [61], as well as an enrichment of IFN-regulated genes. Thus, in SLE the CD21–/low MBCs are seemingly bound to become plasma cells.

Taken together, among the genes expressed by CD21–/low MBCs in healthy individuals and individuals with inflammatory conditions seemingly have in common is *AICDA*. This encodes the AID enzyme that is involved in somatic hypermutation and class-switch recombination, events that take place inside GCs or extrafollicularly [90, 91]. Expression of this gene in circulating cells is, at least to us, rather surprising considering that the activity of this enzyme introduces mutations, albeit at specific sites. The CD21–/low cells also share expression of *TNFRSF1B*, encoding the high affinity TNF receptor that is involved in, for instance, cell survival. Another gene that is shared, at least in most inflammatory conditions, is *SYK*, which encodes a tyrosine kinase involved in BCR signalling. Also shared in some conditions is *TLR7/9* expression, which is consistent with the finding that in several conditions the cells can be activated with TLR agonists in combination with, e.g. cytokines. In this context, the expression of cytokine receptors such as *IL21R* and the lack of *IL4R*, together with the detection of genes regulated by the IL21R, as well as by IFN and IFNγ, are consistent with studies in mice [92]. Here, it was shown that TLR activation in combination with IL21 or IFNγ promotes Tbet expression in CD21−/low B cells and that this expression is inhibited by IL4, whereas CD11c expression is linked to IL21. Genes that might distinguish the CD21−/low MBCs in the various conditions are those linked to plasma cell differentiation, e.g. *PRDM1*, *XBP1,* and *IRF4*, expressed in PB in SLE but not in tonsils in healthy individuals; the status of these genes in the other human conditions is less clear. In this context, we find the study in malaria noteworthy [29], where it was demonstrated how gene expression varied over the course of the disease and in convalescence. It would seem that IL21 and IFNγ are important early in the infection, while a week after acute infection the cells take on a plasma cell precursor signature. This recalls the importance of considering also disease duration and immunosuppressive treatments that can have an impact on the B cells, parameters that may affect the read-out, and even our understanding of the CD21–/low MBCs.

## Ontogeny of CD21–/low memory B cells

In mice, ABCs are thought to develop from mature, naïve (follicular) B cells based on the observation that after the transfer of such cells into congenic hosts, cells with an ABC phenotype were found to be expanded [52]. ABCs express MBC markers indicative of different MBC populations [93], although it is unclear whether they are generated directly from naïve cells or via ‘classic’ MBCs. Nevertheless, the generation of ABCs is dependent on T cells and TLR7 [11, 94], and CD11c+Tbet+ B cells develop in a T-cell dependent manner outside GCs [95].

In humans, generation/maintenance of CD21–/low B cells is dependent on T cells, *IL21R* and Tbet while the importance of TLR is less clear [17, 86]. The CD21–/low cells are antigen-experienced MBCs in the majority of conditions, as the cells are isotype-switched and express BCRs that have undergone somatic hypermutation. As in mice, it is unclear whether these cells are generated directly from naïve B cells, although there are indications that they develop from naïve B cells via MBCs [3]. This could indicate that the cells are formed in GCs; however, class switching and somatic hypermutation can also take place extrafollicularly [91]. In fact, in LN from HIV viraemic patients, CD21–/lowTbet^hi^ MBCs accumulated outside GCs [39], although this does not necessarily mean that HIV is representative of all conditions with an expanded Tbet population.

## Concluding remarks

In all chronic inflammatory conditions discussed above, CD21–/low MBCs have been found to be expanded. In most conditions these cells, or subsets thereof, associated with clinical parameter(s), e.g. in SLE with disease activity, in RA with joint destruction and in malaria with anaemia. Based on the chemokine-receptor expression the PB CD21–/low MBCs in chronic inflammatory conditions would be expected to migrate to sites of inflammation. Indeed, in SLE they are found in kidneys, in RA in inflamed joints, in HIV in LNs, and in Crohn’s disease in the inflamed intestine. The function of CD21–/low MBCs in SLE may well be as plasma cells, as the expressed genes suggest that they are precursors of these cells, and the CD21–/lowCD11c+Tbet^hi^ subset correlates with both plasma cell frequencies and autoantibody levels. In malaria, the CD21–/lowCD11c+Tbet^hi^ correlate with antibodies against red blood cells, and anaemia. Here, plasma cell genes are expressed in convalescence but not the acute phase, indicating that the CD21–/low cells might have different functions at different time points. In most other conditions expression of plasma cell genes has not been reported, either because they were not tested for or they were not detected, except for the study on tonsillar FcRL4+ cells where these genes were not expressed. Therefore, the CD21–/low MBCs in the other conditions are seemingly not precursors of plasma cells, suggesting that they have an, as yet unknown function(s). Whether this unknown function is the same as that of PB in health is currently unknown, but a possibility. Thus, this argues that the CD21–/low B cells are not comparable in the different conditions.

## Data Availability

Not applicable.

## Abbreviations

ABCs: age-associated B cells
ACPA: anti-citrullinated protein antibodies
AID/AICDA: activation-induced cytidine deaminase
BAL: bronchoalveolar lavage
BM: bone marrow
BCR: B cell receptor
BLIMP: B lymphocyte-induced maturation protein
CD: cluster of differentiation
CVID: common variable immunodeficiency
CR: complement receptor
CXCR: chemokine receptor
DAS28: disease activity score 28 joints
DN: double negative
dsDNA: double-stranded DNA
GC: germinal centre
FCRH: Fc receptor homologue
FcRL: Fc receptor-like
HBV: hepatitis B virus
HB: hepatitis B
HCV: hepatitis C virus
HIV: human immunodeficiency virus
IFN: interferon
IFNLR: interferon lambda receptor
IG/Ig: immunoglobulin
IL: interleukin
IL4R: interleukin 4 receptor
IRTA: Immune receptor translocation-associated protein
IRF4: interferon regulatory factor
ITIM: immunoreceptor tyrosine-based inhibitory motif
ITAM: immunoreceptor tyrosine-based activation motif
ITGAX: CD11c/integrin αX
LCMV: lymphocytic choriomeningitis virus
LN: lymph node
MBCs: memory B cells
MS: multiple sclerosis
PB: peripheral blood
P. falciparum: Plasmodium falciparum
PRDM: PR domain zinc finger protein
RF: rheumatoid factor
RNP: ribonucleoproteins
sc-RNAseq: single-cell RNA sequencing
SLE: systemic lupus erythematosus
SLEDAI: Systemic Lupus Erythematosus Disease Activity Index
SLO: secondary lymphoid organs
RA: rheumatoid arthritis
Sw: switched
Tbet: T-box expressed in T cells
TBX: T-box transcription factor
TLR: toll like receptor
TNF: tumor necrosis factor
TNFRSF: tumour necrosis factor receptor superfamily
UnSw: unswitched
XBP1: x-box binding protein.

## Future prospects

In autoimmunity, the CD21–/low MBCs often correlate to disease-specific manifestations, and in chronic infections they contribute to production of disease-specific neutralizing antibodies. Although the knowledge of these cells has exploded over recent years, many questions still remain. For instance, how are the CD21–/low MBCs generated? What is the function of the CD21–/low MBCs in the different conditions? What are their function in the inflamed tissue? Do the cells change function during the course of a disease? Are these cells pathogenic or do they have a protective potential? Can they provide an inhibitory or stimulatory treatment target?
